# P66shc and its downstream Eps8 and Rac1 proteins are upregulated in esophageal cancers

**DOI:** 10.1186/1478-811X-8-13

**Published:** 2010-06-18

**Authors:** Muneesa Bashir, Deeba Kirmani, Hina F Bhat, Rafia A Baba, Rouf Hamza, Sameer Naqash, Nisar A Wani, Khurshid I Andrabi, Mohammad A Zargar, Firdous A Khanday

**Affiliations:** 1 Department of Biotechnology, University of Kashmir, Jammu and Kashmir, India; 2 Department of Biochemistry, University of Kashmir, Jammu and Kashmir, India; 3 Department of Botany, University of Kashmir, Jammu and Kashmir, India; 4 Department of Surgery, Sheri-Kashmir Institute of Medical Sciences, Soura, Jammu and Kashmir, India; 5 Department of Surgery, Government Medical College, SMHS hospital, Srinagar, Jammu and Kashmir, India

## Abstract

Members of Shc (src homology and collagen homology) family, p46shc, p52shc, p66shc have known to be related to cell proliferation and carcinogenesis. Whereas p46shc and p52shc drive the reaction forward, the role of p66shc in cancers remains to be understood clearly. Hence, their expression in cancers needs to be evaluated carefully so that Shc analysis may provide prognostic information in the development of carcinogenesis. In the present study, the expression of p66shc and its associate targets namely Eps8 (epidermal pathway substrate 8), Rac1 (ras-related C3 botulinum toxin substrate1) and Grb2 (growth factor receptor bound protein 2) were examined in fresh tissue specimens from patients with esophageal squamous cell carcinoma and esophageal adenocarcinoma using western blot analysis. A thorough analysis of both esophageal squamous cell carcinoma and adenocarcinoma showed p66shc expression to be significantly higher in both types of carcinomas as compared to the controls. The controls of adenocarcinoma show a higher basal expression level of p66shc as compared to the controls of squamous cell carcinoma. The expression level of downstream targets of p66shc i.e., eps8 and rac1 was also found to be consistently higher in human esophageal carcinomas, and hence correlated positively with p66shc expression. However the expression of grb2 was found to be equal in both esophageal squamous cell carcinoma and adenocarcinoma. The above results suggest that the pathway operated by p66shc in cancers does not involve the participation of Ras and Grb2 as downstream targets instead it operates the pathway involving Eps8 and Rac1 proteins. From the results it is also suggestive that p66shc may have a role in the regulation of esophageal carcinomas and represents a possible mechanism of signaling for the development of squamous cell carcinoma and adenocarcinoma of esophagus.

## Findings

Shc (src homology and collagen homology domain) containing proteins were cloned using an SH2-coding sequence probe and the shc family includes three members with molecular masses 46 kDa (p46Shc), 52 kDa (p52Shc) and 66 kDa (p66Shc). All isoforms are generated either RNA splicing or alternative translational initiation [1]. P66shc has the same modular structure of p46shc/p52shc (SH2-CH1-PTB), however it contains a unique amino-terminal CH2 region, responsible for its distinctive role in signal transduction [2]. Whereas p46Shc and p52Shc are the two cytoplasmic adaptor proteins implicated in the propagation of intracellular signals from activated tyrosine kinases to Ras, p66shc functions in the intracellular pathways involving ROS (reactive oxygen species) generation and apoptosis [1-3].

Unlike p46shc and p52shc, ectopic expression of p66shc is unable to transform mouse NIH-3T3 fibroblasts *in vitro *[2]. Over expression of p66shc protein in cell lines such as Hela, CHO and COS-1 cells does not increase EGF-induced ERK/MAPK activation [2,4]. One possible explanation is that increased expression of p66shc results in elevated level of the basal activity of ERK/MAPK in the absence of stimulus, which thus diminishes additional activation by growth factors [2,3,5]. P66shc is phosphorylated at ser36 in its CH2 domain under various stress signals such as H_2_O_2, _UV radiation and exposure to chemicals, such as Taxol, and thus could serve as an apoptotic sensitizer to stress signals [6,7].

P66shc has also been identified as a mediator of Rac-1 induced oxidative stress. Rac1 is a member of small GTPases that play an important role in regulation of intracellular ROS. Expression of constitutively active Rac-1 increased phosphorylation, reduced ubiquitination and increased stability of p66shc protein [8]. Conversely, p66shc activates Rac-1 through the mediation of exchange factor Sos1 [9]. Eps8 together with E3b1 (another adaptor protein) in a complex are also involved in the regulation of this activity of Rac1 [10]. It has been revealed that Sos1 can either exists bound to Grb2 or Eps8/E3b1. While as Sos-Grb2 complex leads to Ras activation [11], the complex of Sos1/Eps8/E3b1 leads to Rac1 activation [10]. P66shc acts as a switch to dissociate Sos1 from Grb2/Sos1 pool to Eps8/E3b1 pool. This complex formation increases the generation of oxidants through the activation of Rac1 protein [9]. Indeed, during severe oxidative stress, increased binding of p66shc to the activated EGFR and Grb2 occurs. This binding dissociates the Sos1 adaptor protein from the EGFR recruited signaling complex, leading to termination of Ras/MEK/ERK activation [12] (figure representing signal transduction pathway of p66shc protein shown in **additional file **1).The data from the above study further validates its position in signal transduction pathways stimulated by oxidative stress. In spite of this, the role of p66shc in human carcinogenesis remains to be understood evidently.

Several lines of evidences indicate that aberrant expression of p66shc could be implied in various stages of carcinogenesis [13-19]. Furthermore, most of the studies have focused on steroid-regulated cancers without much emphasis on most prevalent cancers existing worldwide. In the present report, we focus on the expression pattern of p66shc and its downstream targets i.e., Eps8 and rac1 proteins in human esophageal carcinomas (one of the most common cancer worldwide). Cancer cells constitutively generate large amounts of ROS suggesting that a certain level of oxidative stress is required for proliferation. Since there is a feedback mechanism between p66shc and ROS generation, and ROS being actively involved in carcinogenesis we sought to look for its expression in cancers as a prelude to its implication for role/target for prevention of tumor progression. Because p66shc regulates Eps8 and Rac1 function by increasing ROS generation, we sought to study their expression pattern along with p66shc.

**Figure 1:**
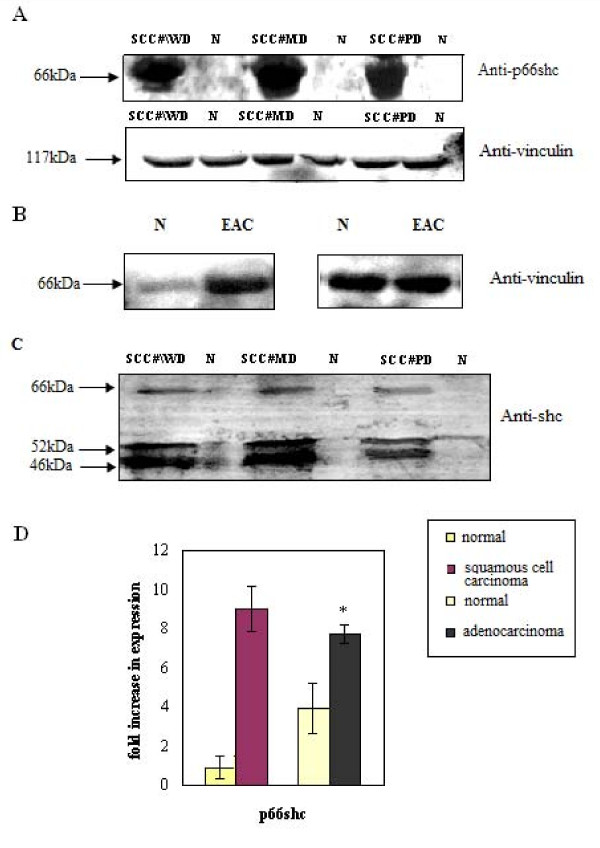
**Expression of p66shc protein in esophageal cancers**. **A **Representative immunoblot showing the up-regulation of p66shc protein in poorly differentiated (PD), Moderately differentiated (MD) and well differentiated (WD) esophageal squamous cell carcinoma with respect to adjacent normals (N). Vinculin polyclonal antibody was used as loading control (Lower Panel). **B **Upregulation of p66shc was also confirmed with antibody which reacts with all the three Shc isoforms. **C **Immunoblot showing the up-regulation of p66shc protein in esophageal adenocarcinoma. Vinculin antibody was used as loading control. **D **Densitiometric analysis of p66shc expression in esophageal squamous cell carcinoma (ESCC) and adenocarcinoma (EAC) as compared to adjacent normals (N). *Columns*, mean of fold increase in expression of four separate experiments, *bars *± SE. * indicates stastically significant (*p *< 0.05) differences compared with adjacent normals as control using *t *test.

We carried out western blot analysis (detailed materials and methods provided in **additional file **2) of p66shc in esophageal squamous cell carcinoma (ESCC) and esophageal adenocarcinoma (EAC) from independent patients (**Table **1). Our results indicate a consistent increase in p66shc protein levels in both carcinomas when compared to their adjacent normal controls (Figure 1). The controls of ESCC showed a lower basal level of p66shc expression (Figure 1A) as compared to the higher levels found in the controls of EAC (Figure 1B). We also carried out expression analysis on well, moderately and poorly differentiated tumor samples. Results indicate (Figure 1A, C) that the increase in p66shc expression as compared to the normal was independent of the different grades of cancers studied. We also used antibody, which recognizes all the three isoforms of ShcA family of proteins. Consistent with the increase in the expression of p52 and p46, we again observed a significant increase in p66shc protein (Figure 1C). Densitometric analysis indicated 4-8 fold increase in the level of expression of p66shc in ESCC as against 2-3 fold increase found in EAC when compared to adjacent normal (Figure 1D). The results obtained were independent of age or sex.

**Table 1:** Table representing distribution of patients and their classification into different cancer grades

Classification of Esophageal cancer	No. of patients	Cancer grade	Sex of patients	No. of patients
Esophageal	65	well differentiated	Male: 25; Female:10	35
Squamous cell		moderately differentiated	Male:12; Female:3	15
Carcinoma(ESCC)		Poorly differentiated	Male: 9; Female:6	15
Esophageal Adenocarcinoma (EAC)	35		Male:24; Female:11	35

It is generally accepted that rapid-growing cells have activated metabolic reactions that lead to increased production of ROS [20]. ROS [e.g., superoxide anion (O_2_^-^), hydrogen peroxide (H_2_O_2_), hydroxyl radical (OH), Peroxy nitrite (ONOO-)] produced by inflammatory and epithelial cells have been implicated in the development of reflux esphagitis, Barrett's esophagus and adenocarcinoma [21]. Based on this report, the p66shc molecule has gained interest, since it is involved in the oxidative stress signaling cascade leading to the production of H_2_O_2 _[6] through p66Shc-Rac1pathway [9]. P66shc acts as a switch to dissociate Sos1 from Grb2/Sos1 pool to Eps8/E3b1 pool. This complex formation increases the generation of oxidants through the activation of Rac1 protein [9]. Conversely, Expression of constitutively active Rac-1 increased phosphorylation, reduced ubiquitination and increased stability of p66shc protein, thereby resulting in further elevation of ROS [8]. This notion is further supported by the observations that raising intracellular ROS level increases the expression of p66shc protein, for example, by H_2_O_2 _treatment [3,22]. It is possible that this signaling mechanism of p66Shc-Rac1-ROS is operative in esophageal epithelial cells leading to carcinogenesis and metastasis.

We also showed that the basal expression level of p66shc in the controls of EAC was higher as against lower levels found in the controls of ESCC. The activity of gastric juices, bile acids and digestive enzymes are more pronounced in lower gastrointestinal tract, which may be responsible for higher physiological values of ROS in EAC [23]. Our tissue study data corroborate our observations on the archival prostrate carcinoma (PCa) tissue specimens that p66shc expression is lower in benign glandular cells, whereas in adjacent adenocarcinomatous cells, the expression of p66shc is higher [16]. Similarly, the expression of p66shc was found to be increased in all kinds of proliferating thyroid tissues, but not in the normal thyroid tissue of the same patient [24]. Collectively, the data support our hypothesis that elevated p66shc protein in esophageal carcinomas plays a critical role in carcinogenesis.

We also observed similar expression of p66shc protein levels in well, moderately and poorly differentiated esophageal squamous cell carcinomas. Since all the samples taken for the study were in stage IIA of cancer development, these results were quite evident. Hence, we are unable to determine if the elevated p66Shc protein level could serve as a diagnostic marker for esophageal cancer progression. However, it can act as a useful prognostic marker for stage IIA of cancers. The clinical application of p66Shc protein in esophageal cancer progression deserves further investigations

**Figure 2:**
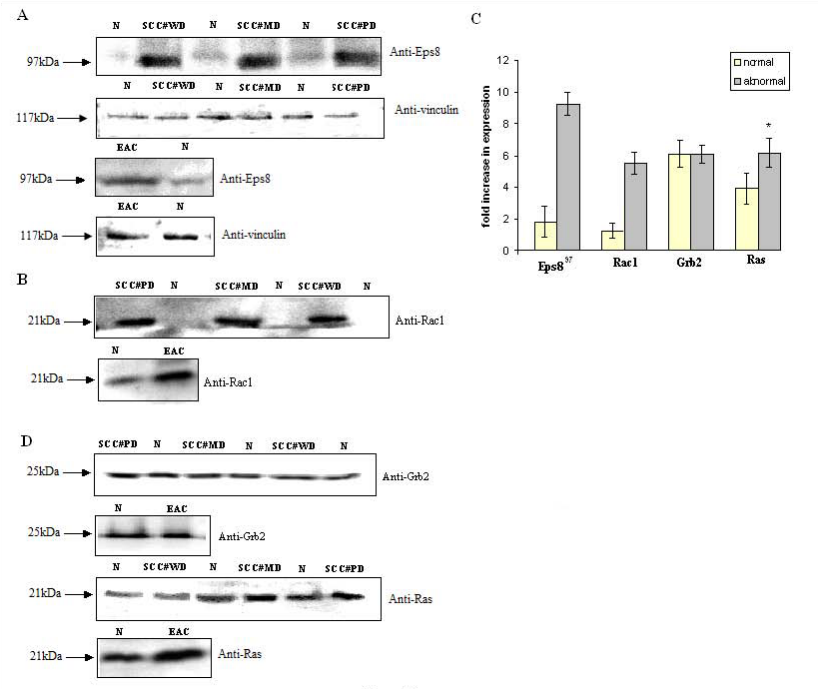
**Expression of eps8, rac1, grb2 and ras proteins in esophageal cancers**. Representative immunoblot showing the elevated levels of expression of **A **Eps8 from poorly differentiated (PD), Moderately differentiated (MD) and well differentiated (WD) esophageal squamous cell carcinoma and esophageal adenocarcinoma samples (lower panel). Vinculin polyclonal antibody was used as loading control. **B **Immunoblot analysis of Rac1, in the PD, MD, WD esophageal squamous and adenocarcinoma. **C **Immunoblot showing the expression of Grb2 and Ras proteins in PD, MD, WD esophageal squamous and adenocarcinoma. **D **Bar chart comparing the expression level of Eps8, Rac1, Grb2 and Ras proteins in esophageal cancers with respect to adjacent normals (N). Values are expressed as fold increase compared to adjacent normal. *Columns*, mean of fold increase in expression of four separate experiments, *bars *± SE. * indicates stastically significant (*p *< 0.05) differences compared with adjacent normals as control using *t *test.

If p66shc participates in the carcinogenesis pathway, then it must be exerting the effect through additional downstream or upstream targets. P66shc is known to promote trimeric complex formation involving Eps8, E3b1 and Sos1, thus leading to an increase in rac1 activity [10]. Hence we were interested in looking at the protein expression level of those critical signaling associates of p66shc which have been previously implicated in regulating cell proliferation i.e., Eps8 [25], and Rac1 [26] regulating the cytoskeletal organization. Results indicate that Eps8 protein expression is higher in ESCC as well as EAC (Figure 2A). We observed a higher level of Eps8 expression in well, moderately and poorly differentiated cancers. The results i.e., higher level of Eps8 expression in tumors as compared to their adjacent normal was independent of the grade of the cancers studied. Our results are consistent with the previously observed studies carried on different types of cancers [25,27,28]. We also observed higher levels of Rac1 protein in cancerous samples relative to adjacent normal samples (Figure 2B). Our densitometric analysis indicated 4-6 fold higher expression of Eps8^97 ^and 3-5 fold increase in expression of Rac1 in esophageal cancers compared to adjacent normal tissue (Figure 2C).

Like p66shc we did find an increase in the basal levels of Rac1 expression in EAC as compared to the ESCC. Rac1 is associated with carcinogenesis and progression of several human tumors [29-31]. Expression of constitutively active Rac1 (Rac1V12) has been found to increase phosphorylation, reduced ubiquitination, and increased stability of p66shc protein. Furthermore, Rac1-stimulated increase in expression of p66shc is dependent on intracellular ROS suggesting that ROS generated by Rac1 activation may lie upstream of p66shc [9]. It is therefore possible that increase in the expression of Rac1 leads to the increase in the expression of p66shc through ROS in esophageal cancers also.

It has been shown that p66shc forms stable complexes with Grb2 [2]. However, during severe oxidative stress, increased binding of p66shc to the activated EGFR and Grb2 occurs. This binding dissociates the Sos1 adaptor protein from the EGFR recruited signaling complex, leading to termination of Ras/MEK/ERK activation [12]. Thus to support the antagonistic activity of p66shc towards growth factor induced MAPK activation, we studied the expression of Grb2 and Ras proteins in esophageal cancers. We observed (Figure 2D) equal expression of Grb2 in esophageal cancers with respect to adjacent normal. We observed an increase in the Ras expression in ESCC and EAC as compared to the normal (Figure 2D). It is possible that the active Ras may even be higher in esophageal cancers as compared to the normal cells. Hence, we do not rule out the possibility of p66shc participating in the development of cancers through Grb2-Ras pathway in addition to operating through Eps8-Rac1 pathway.

Our data support for a novel functional role of p66shc in esophageal cancers. We surmise a positive correlation of p66shc, Eps8 and Rac1 in executing the signal leading to excessive cell proliferation. Although p66shc can be involved in ROS production [9], p66shc may be involved in tumorigenesis through other pathways [32]. It is possible that this relationship between Rac1-mediated increase in p66shc might lead to an increase in expression of Eps8, which eventually might lead to the development of cancer. Hence, determining other upstream effectors and downstream regulators of p66shc may help to identify the mechanisms responsible for cancers and to design strategies for the manipulation of their expression levels *in vivo*.

## Competing interests

The authors declare that they have no competing interests.

## Authors' contributions

**MB: **Preparation of manuscript, western Analysis of p66shc, ShcA, Eps8 adenocarcinoma and statistical analysis of the blots. **DK: **Western Analysis of Grb2. **HFB: **Western blot analysis of Eps8 SCC, Vinculin and Rac1. **RAB: **Western blot analysis of Ras protein. **RH: **Coordinated statistical analysis of the Blots. **SN: **Surgical operation and sample collection from patients. **NAW: **Provided esophageal cancer tissue samples **KIA: **Experimental suggestions and correction of Manuscript. **MAZ: **Experimental suggestions and correction of Manuscript. **FAK: **Designed the work, edited the manuscript, coordinated the group and overall invigilator of the study. All authors have read and approved the final manuscript.

## Supplementary Material

Additional file 1 **P66shc and its downstream Eps8 and Rac1 proteins are upregulated in esophageal cancers**. The figure represents the signal transduction pathway of p66Shc protein. Click here for file

Additional file 2 **P66shc and its downstream Eps8 and Rac1 proteins are upregulated in esophageal cancers**. The data provided represent the Materials and Methods used to carry out the study which include. Specimens, Chemicals, Protein extraction and estimation, Antibodies, Western blotting and Statistical analysis. Click here for file
