# Supplementary figures and images for: P66shc and its downstream Eps8 and Rac1 proteins are upregulated in esophageal cancers
Source: Cell Commun Signal. 2010 Jun 18;8:13. doi: 10.1186/1478-811X-8-13 (PMC2901305; doi:10.1186/1478-811X-8-13)

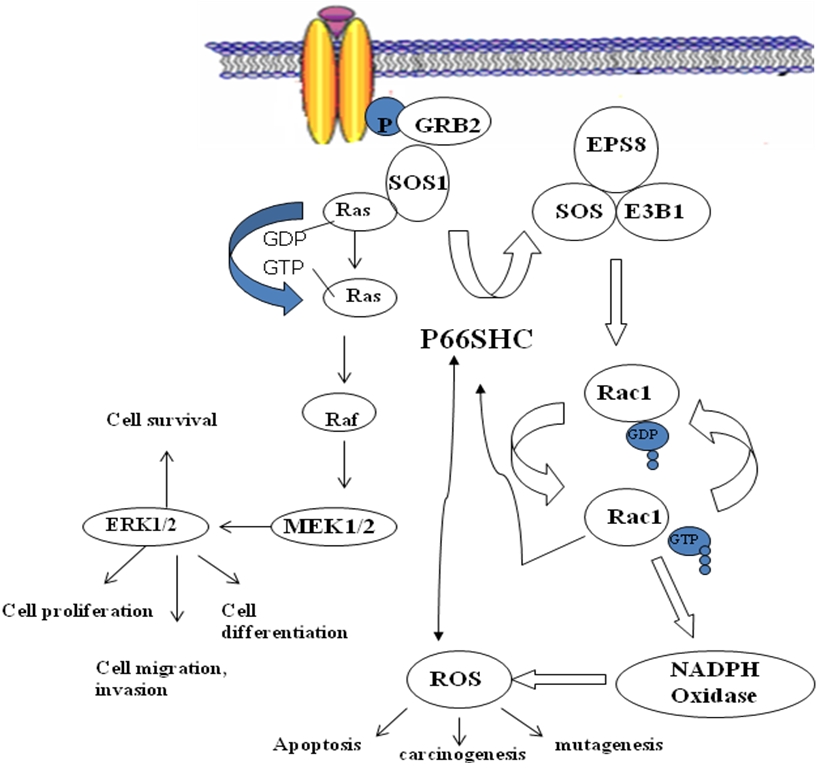


**Figure representing the signal transduction pathway of p66Shc protein**

Supplement: Additional file 1 — P66shc and its downstream Eps8 and Rac1 proteins are upregulated in esophageal cancers. The figure represents the signal transduction pathway of p66Shc protein. [file 1478-811X-8-13-S1.DOC]
