# Supplementary material for: P66shc and its downstream Eps8 and Rac1 proteins are upregulated in esophageal cancers
Source: Cell Commun Signal. 2010 Jun 18;8:13. doi: 10.1186/1478-811X-8-13 (PMC2901305; doi:10.1186/1478-811X-8-13)
Supplement: Additional file 2 — P66shc and its downstream Eps8 and Rac1 proteins are upregulated in esophageal cancers. The data provided represent the Materials and Methods used to carry out the study which include. Specimens, Chemicals, Protein extraction and estimation, Antibodies, Western blotting and Statistical analysis. [file 1478-811X-8-13-S2.DOC]

**MATERIALS AND METHODS**

***Specimens***

Human tissue samples (n=100) of esophageal cancer and their adjacent non-cancerous normal tissues (70 males and 30 females, mean age 58.3 years±5, range 40-70 years) were obtained from the department of CVTS, SKIMS, Soura. J&K, India at the time of surgery. The patients were not given any radiotherapy or chemotherapy before surgery. All the patients have pathological evidence of disease and were in stage IIA of cancer. 65 patients were confirmed as esophageal squamous cell carcinoma (among these patients 35 samples were from patients with moderately differentiated squamous cell carcinoma, 15 samples were each from patients with poorly and well differentiated squamous cell carcinoma) and 35 samples were from patients with adenocarcinoma. The adjacent normal tissues taken for the study were about 3 cm away from the tumorigenic area. Ethical permission, rules and regulations (No. SIMS 186 212/09-2019) were followed for obtaining the samples. All the tissues were frozen within 10 min of removal from the patient. Human tissue samples were placed in sealed cryovials and were subsequently snap frozen by immersion in liquid nitrogen N_2_ (l) and stored in liquid nitrogen N_2_ (l) until use.

***Chemicals***

Bradford micro-protein estimation kit was purchased from Genei laboratories, Bangalore, India. PVDF membrane was purchased from Whatmann. Prestained protein molecular weight markers were from Fermentas. All electrophoresis reagents were obtained from Sigma-Aldrich, Qualigens (Mumbai, India) and Spectrochem (Mumbai, India). All the chemicals for carrying out protein extraction and western blotting were of analytical grade and were acquired from Sigma-Aldrich.

***Protein extraction and estimation***

To 100 mg of tissue 1ml of 0.5% trypsin-EDTA was added and incubated at 37°C for 5min. This was followed by centrifugation at 12,000 rpm for 5 min. The pelleted tissue was rinsed twice with ice-cold PBS, pH 7.4. At all times, the tissues were kept in an ice- bath at 0ºC to prevent warming and potential proteolysis. The tissues were then suspended in ice cold lysis buffer (NP-40 buffer) containing protease and phosphatase inhibitors i.e., 20 mM Tris Cl pH 8.0, 137 mM NaCl, 1% Nonidet P-40, 1% glycerol, 2 mM EDTA, 10 mM NaF, 1 mM PMSF, protease inhibitor cocktail 10μl/1ml of lysis buffer (1000xstock). The lysates were then put on ice for 45 min. This was followed by centrifugation at 12,000 rpm for 10 min at 4ºC. The protein concentration of the supernatant was determined spectrophotometrically (Shimadzu, Japan) at 595 nm with the Bradford’s assay kit as suggested by the manufacturer.

***Antibodies***

The antibodies and their sources were as follows: Antibody to p66shc (Upstate Biotechnology Inc.), Eps8, Rac1, Grb2, and alkaline phosphatase conjugated anti-rabbit IgG (Santa Cruz Biotechnology, Inc.) were purchased. Shc A monoclonal antibody (Trans Lab), Vinculin polyclonal antibody, total Ras monoclonal antibody (Sigma) and alkaline phosphatase conjugated anti-mouse IgG were gift from S. Andrabi (Harvard Medical School).

***Western blotting***

A volume containing 40 μg of protein extract was heated to 100°C for 5 min in reducing sample buffer containing 50 mM Tris Cl (pH 6.8), 2% SDS, 10% glycerol, 0.1% bromophenol blue, 100 mM β-mercaptoethanol and loaded onto 10% SDS-polyacrylamide gel. Proteins were then transferred electrophoretically to PVDF membrane at 80 V for 2 hrs at room temperature. Membranes were subsequently immersed in 0.1% commassie brilliant blue for staining and to conform that equal amounts of protein were loaded in each lane and transferred efficiently. The PVDF membranes were incubated for 1.5 hrs in a blocking solution containing 5% NFDM in TBS, pH 7.4. The membranes were incubated at room temperature for 2 hr with a primary antibody (1:1000 dilution of anti-p66shc Ab, 1: 400 dilution of anti-Rac, 1:400 dilution of anti Grb-2, 1:400 dilution of anti-Eps8 and 1:500 dilution of anti-vinculin, 1:1000 dilution of shcA and 1:500 dilution of Ras). Membranes were washed three times in TBS supplemented with 0.1% Tween 20 and incubated for 1 hr with alkaline phosphatase conjugated anti- rabbit IgG (1: 3500) and anti-mouse IgG (1:1000) in TBS. The blots were then washed again and antibody binding was visualized by using BCIP/NBT chromogenic substrate. Quantitation of the bands was carried out by densitometric analysis using quantity one (4.5.0) 1D analysis software.

***Statistical analysis***

Experiments were performed in triplicate or quadruplicate, and results were calculated as mean ± S.D. Statistical analyses were performed by *t* test (Microsoft Excel), and p < 0.05 was considered statistically significant.
